# Expanded Polystyrene/Tyre Crumbs Composites as Promising Aggregates in Mortar and Concrete

**DOI:** 10.3390/polym16223207

**Published:** 2024-11-19

**Authors:** Karamat Subhani, Krishnamurthy Prasad, Nishar Hameed, Mostafa Nikzad, Nisa V. Salim

**Affiliations:** School of Engineering, Swinburne University of Technology, Hawthorn, VIC 3122, Australia

**Keywords:** recycling, sustainability, expanded polystyrene, granulated tyre rubber, blending, mortar, concrete

## Abstract

A composite material comprising expanded polystyrene (EPS), granulated tyre rubber (GTR), and a compatibilizer is demonstrated as a possible replacement for fine and coarse agglomerates in mortar and concrete systems, respectively. Two different polymer blending processes (solvent/low shear blending and melt/high shear blending) are used, and the resulting composite material utilized as aggregate to replace sand and cement for mortar and concrete block development. Critical properties such as workability, compressive and flexural strengths, water absorption, bulk density, and porosity are measured before and after aggregate replacement. The novel composite material led to significant improvements, boosting compressive strength by 7.6% and flexural strength by 18% when sand was replaced and further increasing compressive strength by 22.2% and flexural strength by 5.26% with cement replacement. However, a decrease in compressive and flexural strength was observed when plain EPS and plain GTR were used separately as aggregate replacements. This work proposes a pathway for the successful reincorporation of difficult-to-recycle materials such as EPS and GTR, otherwise destined for landfill, back into the supply chain for the construction industry. Moreover, this research represents the first reported work where the overall properties of mortar have surpassed those of standard mortar when substituted with recycled EPS or GTR.

## 1. Introduction

The rapid increase in global population over the past few decades has greatly enhanced the demand for raw materials [1]. This high demand has consequently increased the raw material cost [2], thus necessitating a sustainable material solution. An optimal solution for this crisis is to utilise waste material from other industries as raw material in the construction industry, thereby achieving the dual outcome of waste recycling the waste/closing the loop and addressing the raw material demand [3]. The prime example can be the use of resilient and environmentally friendly structural elements for the construction industry [4]. This includes the use of geopolymers [5], recycled steel [6], bamboo [7], hemp [8], and more importantly, recycled plastic composites [9]. However, it is very challenging to achieve properties comparable to existing materials using raw waste materials [10].

Thermoplastics such as Polystyrene (PS), Polypropylene (PP), and Polyethylene (PE) are among the largest parts of landfill waste worldwide [11,12,13]. PS. in its expanded form (EPS) is widely used in the packaging/insulating industry and is difficult to effectively recycle due to its brittle nature and very low weight-to-volume ratio [14]. Per estimations conducted in [15], 14 million tons of EPS are produced worldwide annually, with most of the EPS produced going to landfills, leading to serious environmental concerns. Recently, there has been a concerted effort [16] to use this EPS waste for construction applications due to its inherent high thermal insulation, low weight, high durability, and low moisture absorption properties [17]. For instance, Menezes et al. replaced 20–40% of sand with EPS to develop mortars and studied physical, thermal, and mechanical properties. They managed to enhance water absorption, but a significant drop of 50% in compressive strength was observed [18]. In another study, A. Milling et al. explored the full replacement of cement with EPS paste in acetone as binder. Compressive strength decreased massively from 10.96 MPa to 0.9 MPa, but was enough for masonry construction [19]. However, these two studies show a significant drop in compressive strength, which can be due to the EPS’s inherent high porosity and brittle nature [20].

Another material that has been explored for a similar purpose is granulated tyre rubber (GTR). GTR waste management is another big issue currently facing the world due to the increasing usage of automobiles. Around 1.5 billion tyres are being produced annually around the globe [21]. Landfilling discarded tyres can postpone the issue but raises problems for the environment in the future [22]. Using GTR waste in the construction industry possesses a lot of potential, and researchers are focusing on resolving the challenges [23]. Particles/crumbs of GTR have natural elongation and can provide the structure with some required flexibility [24]. One of the biggest challenges is addressing the interfacial interaction between the relatively hydrophobic GTR and the relatively hydrophilic materials comprising civil raw materials [25]. The interfacial tension and consequent low adhesion reduce the workability and compressive strength of the fabricated civil structure. Recently, M. Sambucci et al. replaced 50% sand with GTR to develop lightweight concretes. Flexural strength and compressive strength dropped significantly, which was due to the low interfacial interaction of DTR with cement [26]. A relatively facile way to reduce interfacial tension is to treat the surface of the GTR [27], but that itself reduces the inherent properties of the GTR and further adds a step to the overall process, thereby reducing the process economy.

There have been several efforts to incorporate both EPS and GTR in concrete and mortar structures. The previous literature suggested that much of the work uses the EPS and GTR separately, with no blending being conducted. A more convincing methodology is to fabricate a composite aggregate that can enhance the interactions between GTR and civil raw materials while still maintaining the overall mechanical/ductile performance of the GTR [28]. By fabricating a composite comprising EPS and GTR, potential material synergy can be created whereby the interfacial interaction can be improved and the inherent brittleness of the EPS can be reinforced.

Also, most of the work uses both the GTR [29] and EPS [30] as replacements for sand [31], but there are examples where the EPS [32] and GTR [33] replace coarse aggregate (gravel) and some rare examples where cement is replaced [34]. Mostly, the use of EPS and GTR as aggregate generally results in a net reduction in compressive and flexural strength; there are works wherein this drop is not prohibitively high (being of the order of 10% as compared to the control specimen) [31]. However, the porosity and water absorption trends are a bit more unpredictable, as both an increase and a reduction are possible depending on the system explored.

Considering the definite lack of work in using a blend/composite of EPS/GTR as an aggregate material for mortar/concrete in the available literature, we have, in this work, successfully developed a composite aggregate comprising EPS and GTR. The effective reinforcement of EPS with the GTR phase and, therefore, an attempt towards generating material synergy is aimed at. In this regard, a thorough analysis of the properties developed in both mortar and concrete is conducted, and the case for more sustained use of the developed composite aggregate is presented. For the first time, using EPS and GTR, we have improved compressive and flexural strength without any treatment to the raw material. This paper demonstrates that blending EPS and GTR can help in reinforcing mortar and concrete structures. All existing literature uses either plain EPS or plain GTR, and in both cases there seems to be an overall trend towards property reduction. In our work, there is a categorical improvement in properties without sacrificing on other critical facets such as workability and bulk density, amongst others.

## 2. Materials and Methods

### 2.1. Raw Material Details and Compositions Made

**Figure 1:**
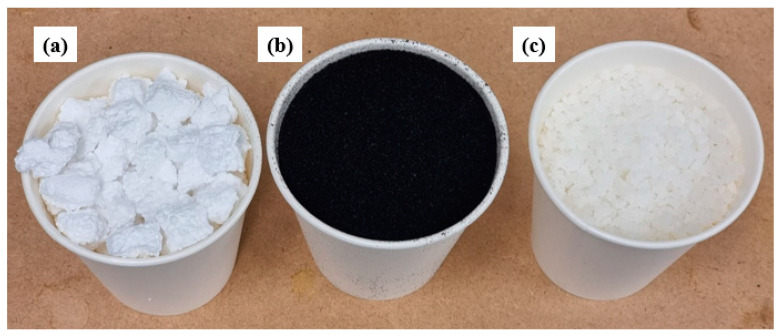
(**a**) Recycled EPS, (**b**) GTR, (**c**) SBS compatibilizer.

Recycled expanded polystyrene (EPS) was sourced in the form of shredded foam pieces (Figure 1a) with a density of 0.026 ± 0.01 g/cm^3^ from Polyfoam Australia Pty Ltd. (Dandenong, VIC, Australia) Frubber Pty Ltd. (Mount Waverley, VIC, Australia) provided the granulated tyre rubber (GTR; Figure 1b) with a 300–600 μm particle size range and a density of 0.50 ± 0.01 g/cm^3^. Styrene-Butadiene-Styrene (SBS; Figure 1c) block copolymer (a compatibilizer) with a density of 1.04 g/cm^3^ was supplied by Merck Australia. Acetone and Toluene (≥99.5%) were also obtained from Merck Australia for solvent blending of the samples. General-purpose cement, fine-washed sand and coarse aggregate were purchased locally from C. Fulton Pty Ltd., Cheltenham, Victoria, Australia, and marble was purchased from Barossa Quarries, South Australia, Australia.

### 2.2. Fabrication Methods

Two different methodologies were used to develop composite aggregate mixture: solvent blending and melt blending.

#### 2.2.1. Melt Blending

**Figure 2:**
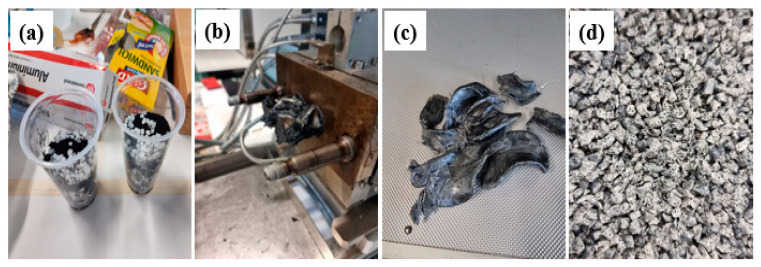
(**a**) Dry mixing of EPS, GTR and SBS (**b**), batch mixing (**c**), output dough of batch mixing, (**d**) shredded material.

In melt blending, pre-melted EPS, GTR, and SBS were first mixed with the specific ratio (Table 1). The mixed raw material was then fed in a Haake PolyLab QC 3′400V/N/PE 32A (Filabot, USA) for 12 min at 190 °C using 60 rpm rotor speed (Figure 2a–c). The dough made, as shown in Figure 2c, was then shredded using a Wittmann MAS1 granulator (Vienna, Austria) to reduce the size of the composite aggregate (M; Figure 2d).

#### 2.2.2. Solvent Blending

**Figure 3:**
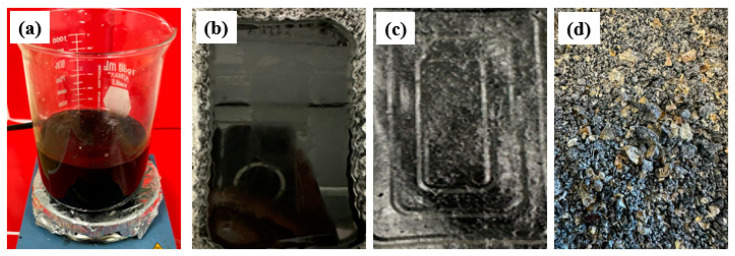
Different stages of solvent blending of EPS with GTR: (**a**) Solution mixing, (**b**) Composite solution for drying, (**c**) Cured composite, (**d**) Shredded material.

In solvent blending, firstly, the 70 wt% of EPS was dissolved in toluene at room temperature. After dissolving, acetone solvent was added. The weight ratio of acetone/toluene was 70:30. The 30 wt% GTR was then added in solution to complete the composite mixture. The mixture was then constantly stirred for 2 h at room temperature (Figure 3a). Afterwards, the mixture was poured onto Al foil trays and dried at room temperature for 48 h (Figure 3b–c) and crushed into smaller-size aggregate (see Figure 3d).

#### 2.2.3. Mortar and Concrete Mixtures

**Figure 4:**
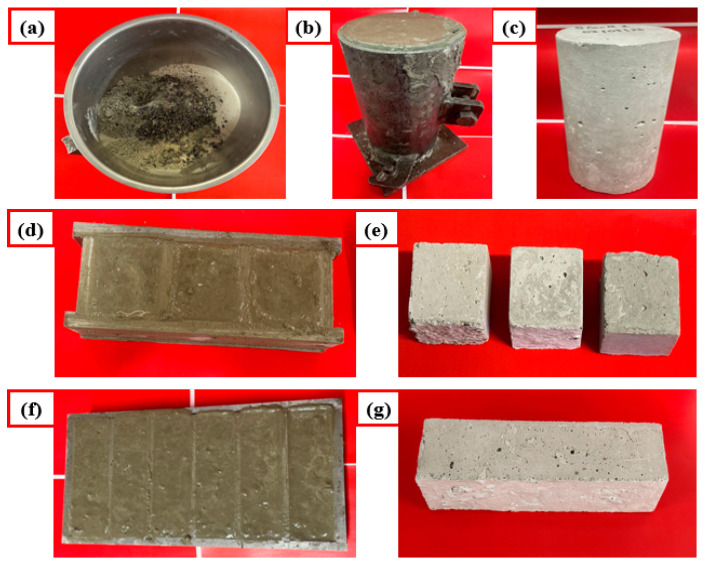
(**a**) Dry mix of the mortar constituents viz., cement, sand, composite aggregate and water, (**b**) cylinder mould for fabrication of concrete specimens, (**c**) concrete cylinder obtained post demolding and 28 days curing in water, (**d**) blended mortar cast into cube moulds, (**e**) cubes obtained post demolding and 28 days curing in water, (**f**) rectangular prism mould for fabrication of flexural testing specimens, (**g**) flexural testing specimen obtained post demolding and 28 days curing in water.

**Figure 5:**
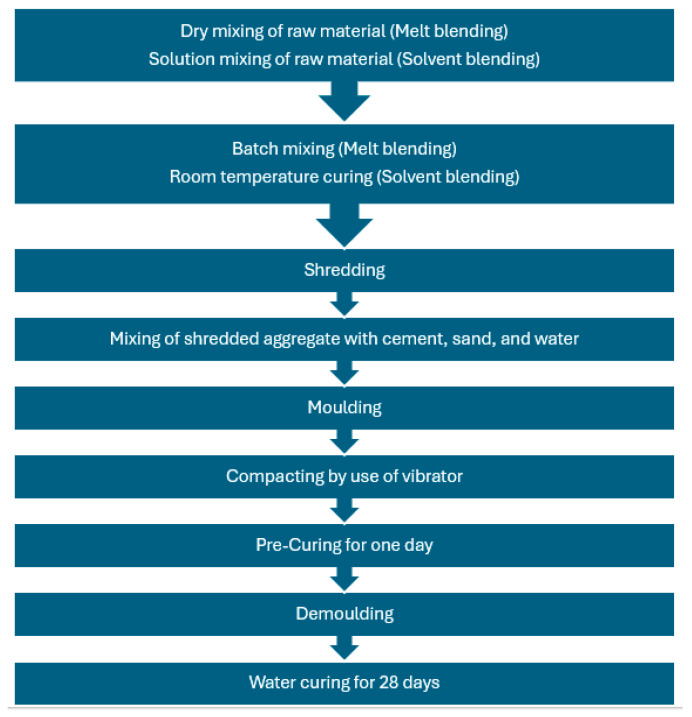
Flowchart of experimental setup.

Two different types of mortar and one concrete mixture were used for investigation. The control specimens were prepared first for reference, and then 10% of the sand/cement was replaced by solvent and melt-blended aggregate (Table 2 and Figure 4 for sample details). The reasoning behind the replacement of sand and cement by waste plastic is the substantial environmental benefits in terms of preventing sand over-dredging, as shown in [35], and remediation of greenhouse gas emissions, as shown in [36]. In all samples, raw material and aggregate were mixed in a planetary mixer (Hobart N50 3-speed, C-2251, Tianjin, China) for around 5 min. Water was poured into the mixture during mixing. A perfectly blended paste was cast in steel moulds and placed at room temperature (24 h) for hardening. After 24 h, samples were demoulded and were cured in water for 28 days for curing (see Figure 5).

### 2.3. Characterisation

**Figure 6:**
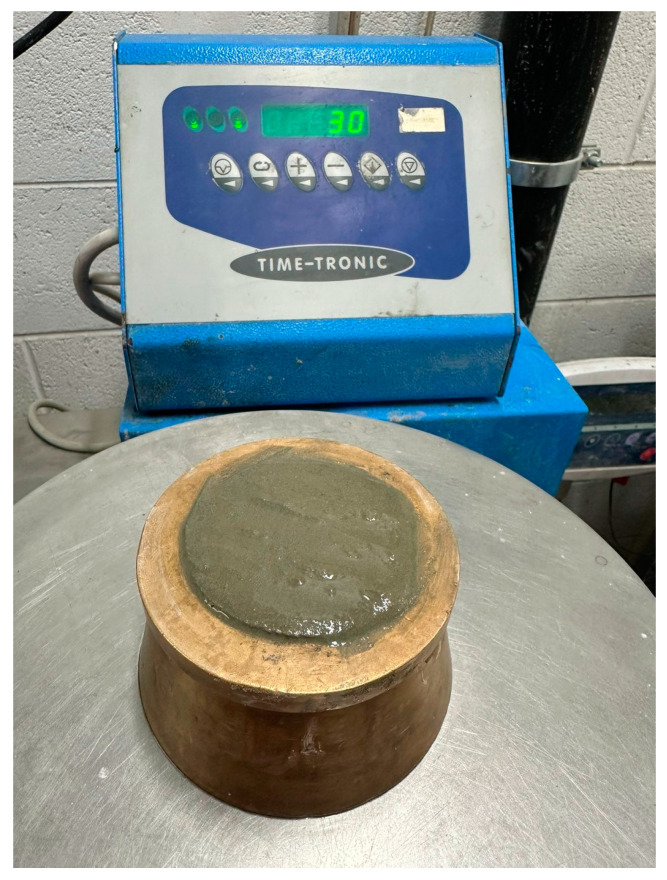
Workability setup with packed slump cone.

The workability of the mortar compositions shown in Table 3 was measured on a Time Tronic^TM^ rotating/vibrating platform (Manufactured by Vtech, Treviolo, Italy) using the slump cone method (setup in Figure 6). The mixtures were mixed on the Hobart N50 mixer (Tianjin, China) for 5 min and then filled into the slump cone. After packing in the mortar and removing the slump cone, the vibrating platform was activated for 30 repetitions. Workability was measured per standard ASTM C230 as the difference in diameter of the slumped mortar pre (100 mm) and post vibration.

**Figure 7:**
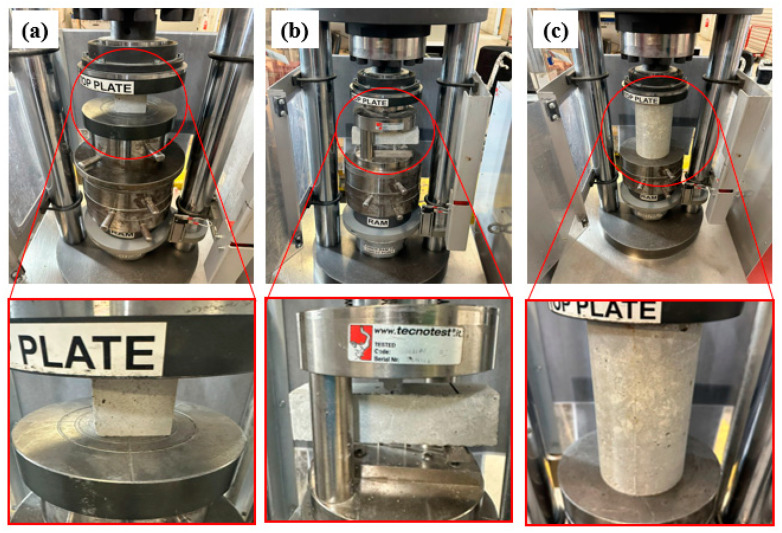
Setup for (**a**) Compression testing of mortar/concrete cubes, (**b**) Flexural testing of mortar/concrete cubes, (**c**) Compression testing of concrete cylinders.

Cubes of 50 mm × 50 mm × 50 mm were cast and cured for 28 days from all compositions detailed in Table 2. These cubes measured properties such as capillary water absorption, compressive strength, porosity, and density (in triplicate). Capillary water absorption coefficient (CWAC) was measured by periodically measuring the increase in mass of the cube samples after immersion in water. The mass uptake per unit area of the samples was then plotted against the square root of the time in min, and the slope of the graph was presented as the CWAC in kg/m^2^/min^0.5^. The compressive strength of the cube and (for concrete specimens) cylinder samples were measured on a Techno^TM^ mechanical testing machine per AS 1012.9: 2014 standards (Figure 7a,c). In addition to the 50 mm × 50 mm × 50 mm cubes, prisms of 250 mm × 50 mm × 50 mm were prepared to perform flexural testing using the setup shown in Figure 7b per AS 1012.11: 2014.

The Porosity (P) of the cast cubes was measured using Equation (1) [37].

(1) $$P=\left[\frac{w_{sat}-w_{d}}{w_{sat}-w_{s}}\right]\times 100$$

**Figure 8:**
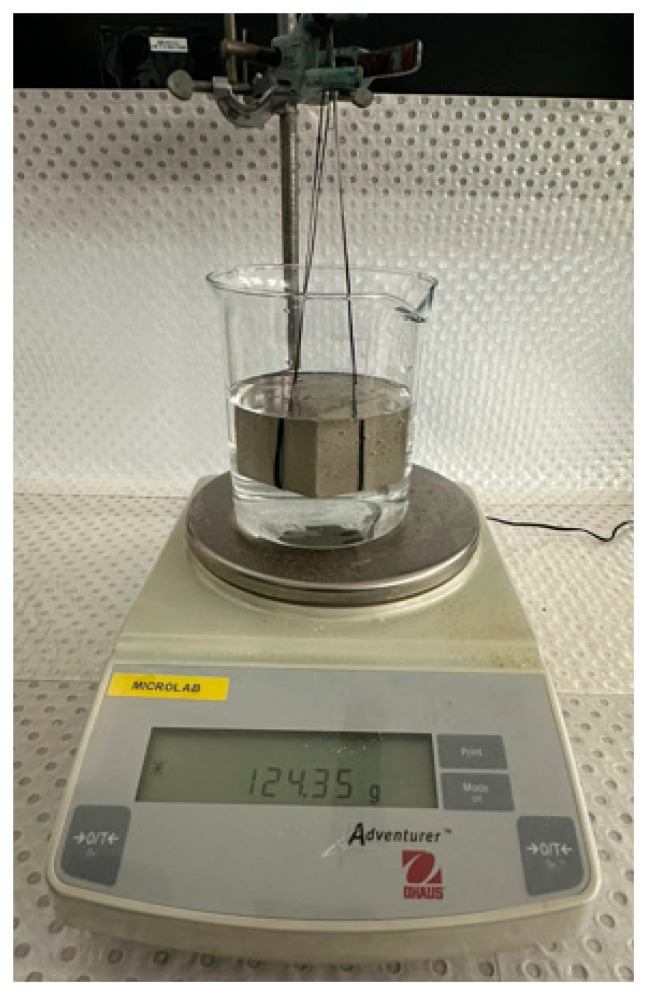
Setup for measurement of sample porosity.

In Equation (1), w_sat_ is saturation weight obtained post-curing after 28 days in water, w_d_ is dry weight measured after drying the cubes in an oven overnight at 110 °C, and w_s_ is suspended weight. The overall setup for measuring the suspended weight is shown in Figure 8. Further, using w_d_, the bulk density of the mortar was measured.

## 3. Results

### 3.1. Workability

**Figure 9:**
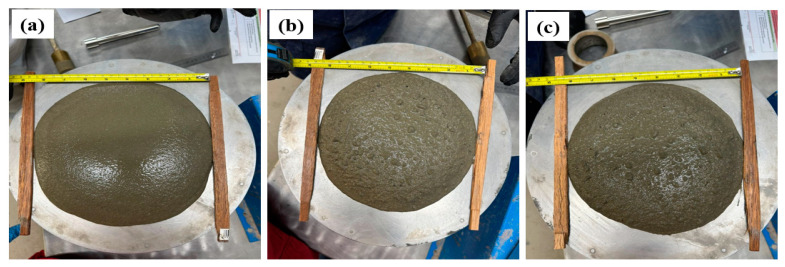
Workability measurements of mortar compositions. (**a**) Control specimen post measurement. (**b**) Melt-blend specimen post measurement. (**c**) Solvent-blend specimen post measurement.

The workability results of the mortar specimens are shown in Table 3, and images of the testing are shown in Figure 9. The control sample possessed a workability of 115 ± 5 mm, which is ideal for a mixture in civil applications [38]. All composite aggregate samples, including M1, M2, S1, and S2, show workability from 105 to 110 mm, which is a little less than control but still within the required range. These results show that the inclusion of composite aggregate does not significantly influence the workability.

### 3.2. Bulk Density and Porosity

The bulk density and porosity of control and composite aggregate-based mortar samples are shown in Table 3. Porosity plays a critical role in the bulk density of mortar samples. Control samples had a bulk density of 2.032 ± 0.004 g cm^−3^ with a porosity of 8.85%. The 10% sand replacement samples (both solvent and melt blended) almost show an identical bulk density, as shown in Table 3. The reported bulk density is 10% less than control samples, which can be due to a 100% increase in porosity [37]. The increase in porosity can be due to the size difference in sand particles and composite aggregate. Sand particles failed to cover spaces due to a 10% quantity reduction. On the other hand, in cement replacement, more sand particles are present to cover the empty spaces in mortar. However, it failed to cover all empty spaces due to a 10% reduction in cement particles. The increase in porosity was reduced to only 10% in the case of cement replacement, which is why the density of cement replacement samples was reduced to only 4%. These results proved that sand replacement had more impact on the weight of the mortar and made the samples lightweight. Moreover, both sand and cement replacement samples were lightweight, which is crucial for the civil industry.

### 3.3. Water Absorption

**Figure 10:**
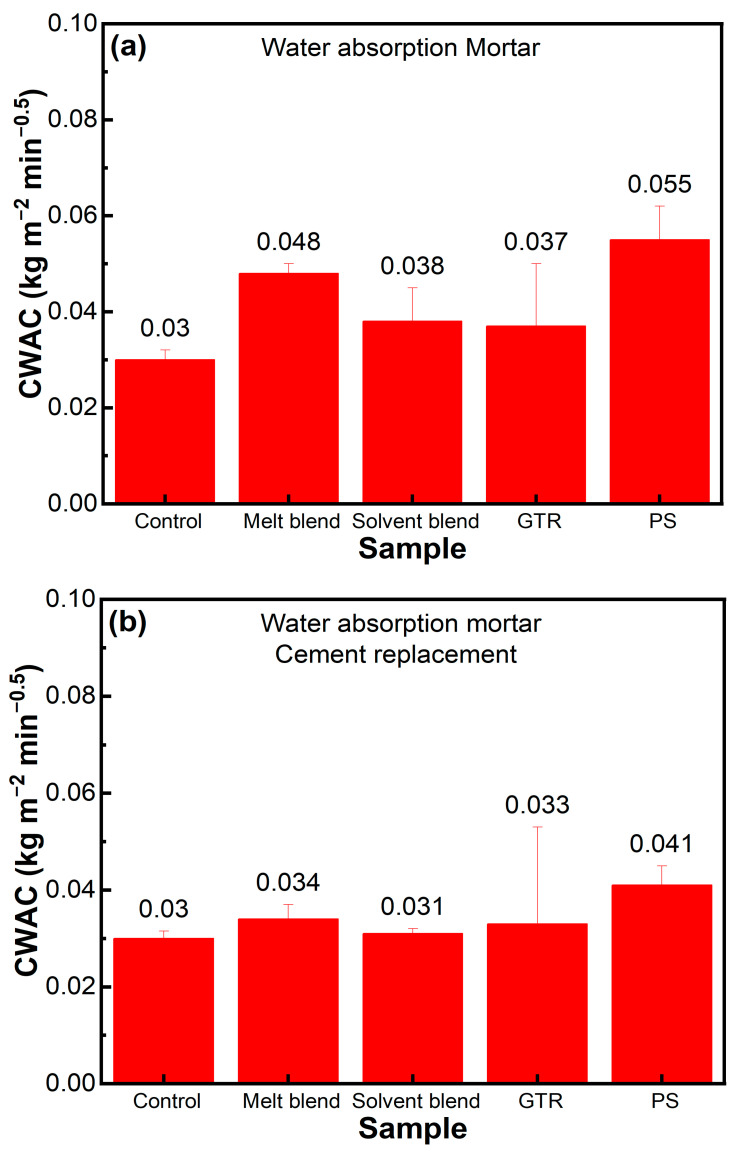

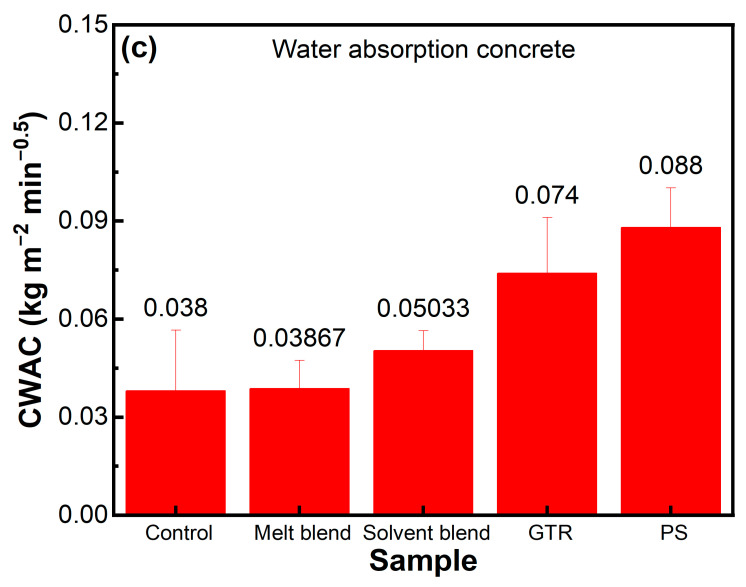
Water absorption of (**a**) sand replacement mortar, (**b**) cement replacement mortar, and (**c**) aggregate replacement concrete.

Figure 10a shows that the water absorption increased with 10% sand replacement by the composite aggregate. This increase in water absorption is due to a high increase in porosity, as shown in Table 3. M1 possessed more porosity, which resulted in an increased water absorption than S1. Similar trends were observed in cement replacement mortar, but the percentage increase is not as much as sand replacement due to an average increase in porosity (see Figure 10b). These results show that both open and closed pores are present in the aggregate-based mortars, providing water droplets a pathway to move within. The number of pores increased in aggregate-based concrete due to variations in coarse aggregate and composite aggregate size, which increases porosity. More porosity leads to high water absorption (see Figure 10c). In concrete, solvent blends possess more water absorption, which is due to the higher size variation. Melt blended composite is more consistent in size.

### 3.4. Compressive Strength

**Figure 11:**
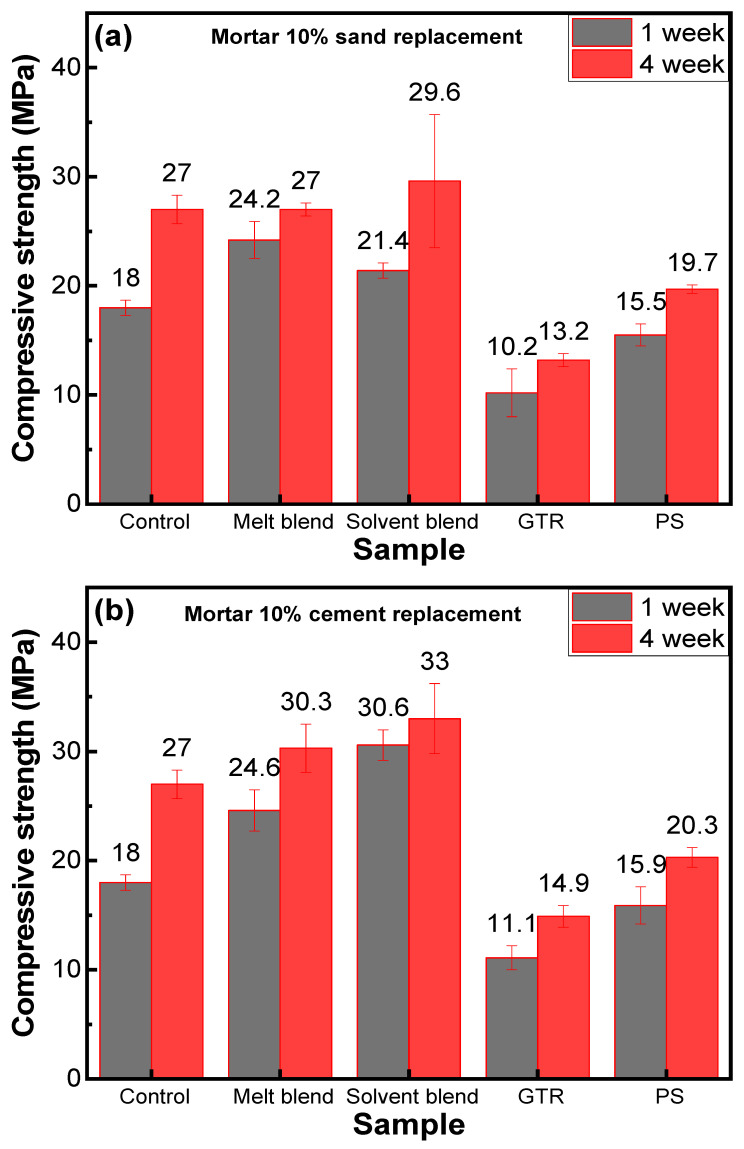

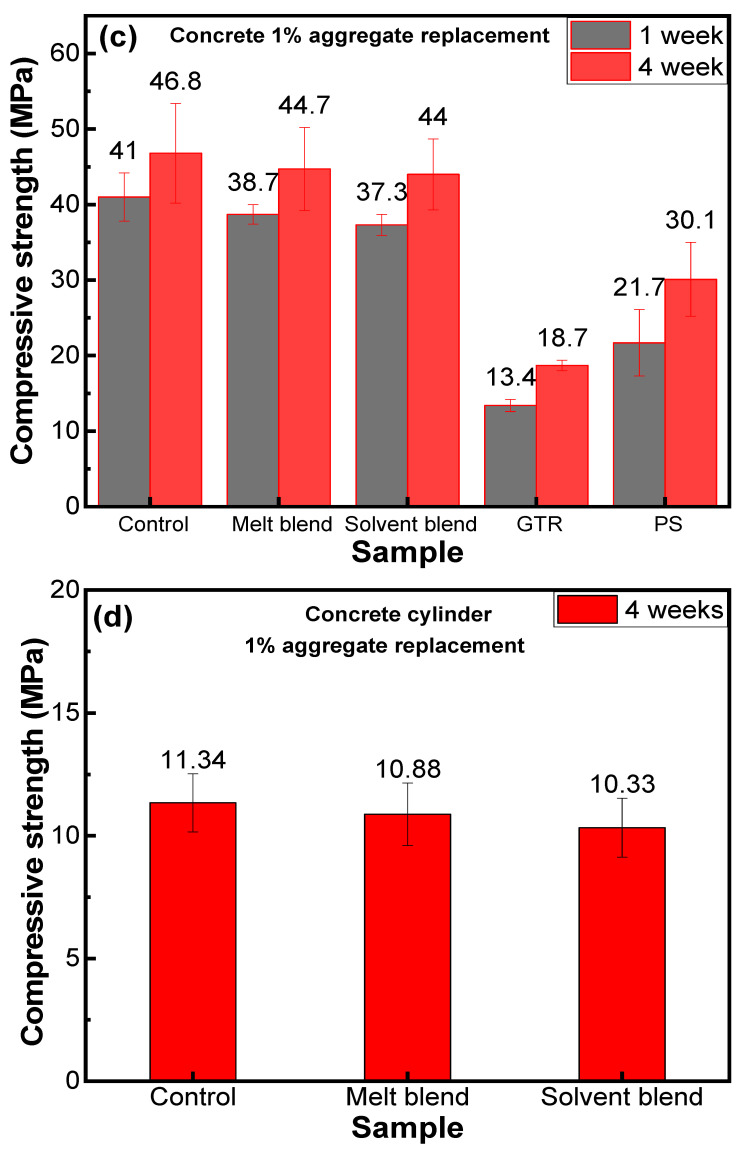
Compressive strength of (**a**) 10% sand replacement mortar, (**b**) 10% cement replacement mortar, (**c**) 1% aggregate replacement in concrete cubes, and (**d**) 1% aggregate replacement in concrete cylinders at different curing periods.

Compressive strength was measured after seven days and 28 days of curing for all samples, including sand replacement mortar, cement replacement mortar, and aggregate replacement concrete, to study the effects of composite aggregate inclusion in mortar/concrete fabrication. Four samples were prepared besides the control sample for sand and cement replacement mortars. The 10% sand replacement compressive strength values are shown in GTR-based mortar, which exhibits lower compressive strength than control samples, which can be due to the very low interface interactions between GTR particles and cement binder (see Figure 11a) [39]. EPS-based mortar also possesses lower compressive strength than control, but the reduction in strength is about 27%, which was more than 50% in the case of GTR. It is evident that EPS shows better interface interactions with cement binders than GTR. However, a 27% reduction in strength can be related to the brittle nature of EPS [17]. Due to these contrasting problems of GTR and EPS, we produced the idea of mixing these two materials together so GTR can reduce the brittle nature of EPS and EPS can hold the GTR together and provide the necessary interface interaction with the binders. Two different techniques, melt blend and solvent blend, were used, and the compressive strength of these two samples was recorded against control samples. M1 shows a similar compressive strength of 27 MPa, and S1 shows a 10% improvement (29 MPa). The 10% cement replacement mortar samples also show a similar trend. However, compressive strength increased from 5 to 10% for all samples from sand replacement samples. M2 shows a higher compressive strength of 30.3 MPa from M1, which can be due to the low porosity of M2 samples (see Figure 11b) [40]. These results prove our theoretical idea that the synergetic effect of EPS and GTR blends can improve the mechanical properties of mortars.

The results in Figure 11c,d show that in concrete (cube/cylinder), there is only a 4% reduction in strength due to 1% coarse aggregate replacement with composite aggregate. This reduction in strength was less than expected because it is very hard to replace coarse aggregate. This shows that composite aggregate can replace 1% coarse aggregate with sand and cement.

### 3.5. Flexural Strength

**Figure 12:**
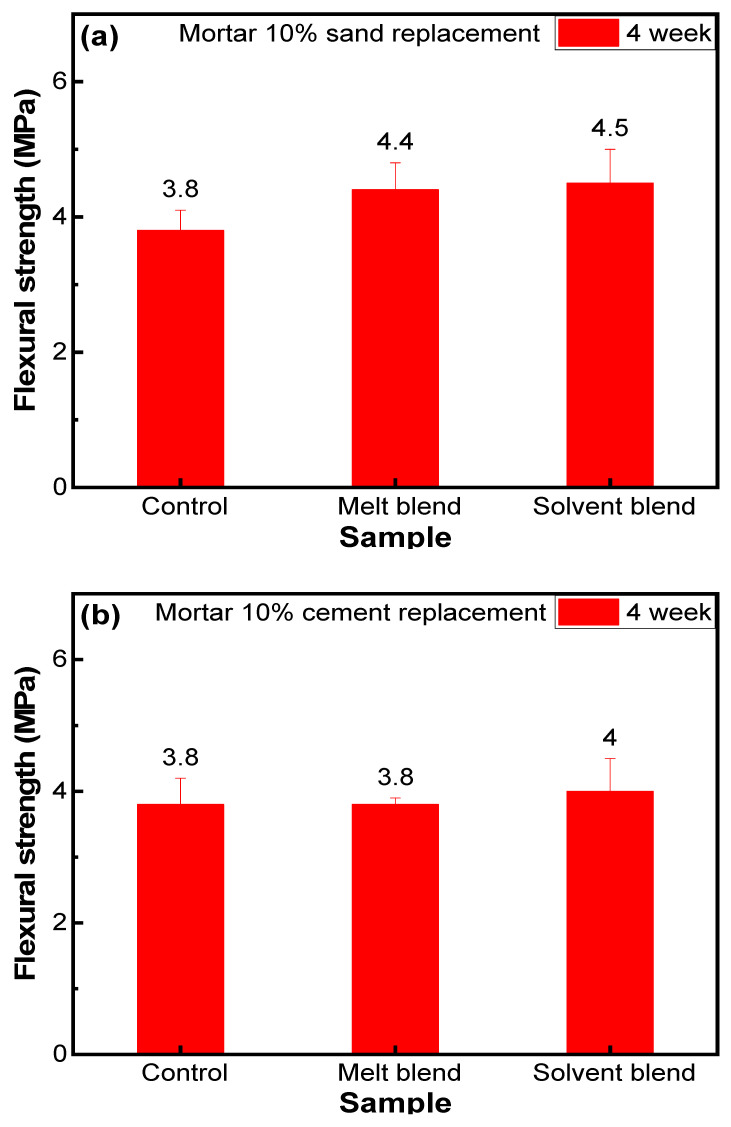
Flexural strength of (**a**) 10% sand replacement and (**b**) 10% cement replacement mortars.

The flexural strength of both sand replacement and cement replacement was recorded and compared with the control to further verify the effects of composite aggregate on the mechanical properties of mortar. Figure 12a shows that 10% sand replacement by prepared aggregate M1 increased the flexural strength from 3.8 MPa to 4.4 MPa, which is almost a 16% enhancement. S1 shows even more enhancement (18.5%) from the control sample, which is quite high compared to the expectations. With the increase in porosity from 8% to almost 18%, the expectations were that M1 and S1 would lose some flexural strength. However, flexural strength increased significantly. This shows that our composite aggregate acts as a reinforcement material and increases the interface interactions between aggregate and cement, as mentioned before.

In contrast, 10% cement replacement provides almost the same flexural strength for the melt blend and a little increase of 5% for the solvent blend, which is understandable (see Figure 12b). Large cement replacement reduced the binding material, so interface interactions are not as strong as in sand replacement/control samples. However, composite aggregate by both methods still provides enough stability that M2 and S2 hold almost equal flexural strength to the control samples.

Moreover, composite aggregate inclusion decreased the brittleness of mortar samples, which was clearly observed during flexural testing. Control samples were brittle and broken straightway into two pieces under loading, while composite aggregate-based mortar samples did not exhibit brittleness.

## 4. Discussion

It is clear from the results and literature (see Table 4) that compressive and flexural strength decreased by replacing sand/cement/aggregate with GTR or EPS, which can be due to the following reasons:

1. GTR possessed very low interface interactions with civil aggregate and raw materials due to availability in the volcanized form. Many researchers used acid/chemical treatments to increase the interface interactions but ended up decreasing the GTR strength.
2. EPS shows a brittle nature, which affects the mortar/concrete compressive strength. Moreover, the high porosity of EPS increased the porosity, which resulted in lower compressive/flexural strength of mortar/concrete samples.

Many researchers focused on improving these properties of GTR and EPS to obtain more compressive and flexural strengths but failed to increase them from control samples. However, we mixed these two waste materials with two different methodologies and developed a composite aggregate in which EPS provides the required interface interaction with the cement and GTR provides the required flexibility (see Figure 12). Both of our composite aggregates managed to increase both compressive and flexural strength from the control samples, as shown in Table 4. It is also clear that future research in this area has to deal with increasing the level of replacement. While in this work, an aggregate replacement level of 10% in mortar and 1% in concrete has been demonstrated to show improvements in mechanical performance, a more categorical development would entail replacement of whole or half of the conventional aggregates, i.e., all future research needs to be focussed on 50% or 100% replacement of the fine and coarse aggregates with the EPS/GTR/SBS composite developed in this work. Also, it has to be noted that while the solvent-assisted blending of composite showed slightly higher mechanical performance when used as the aggregate replacement, its prospects as a sustainable and green alternative are reduced owing to the use of volatile organic solvents (acetone and toluene). In terms of both sustainability and scalability, the melt blended approach seems to possess higher potential.

## 5. Conclusions

This work explores the recycling of two difficult-to-recycle materials, such as EPS and GTR, in the construction industry. A review of the literature shows that the EPS and GTR may be used separately and can achieve significant weight reduction for both mortar and concrete structures. However, the structural properties, including compressive and flexural strength, are compromised. To counter this, an effective blending strategy was developed that could achieve synergy by combining the individual positive structural attributes of EPS and GTR. Two different blending methodologies, viz., solvent and melt blending, were considered, and the composite aggregate thus fabricated was then used in mortar and concrete. Irrespective of the blending technique, we managed to improve the compressive strength and flexural strength of both 10% sand (7.6% increase in compressive strength and 18.4% increase in flexural strength) and cement replacement (22.2% increase in compressive strength and 5.26% increase in flexural strength) mortar samples for the first time without any pre-treatment of the raw materials. Additionally, the bulk density of all the aggregate-replaced samples was reduced, confirming that composite aggregate decreases the structural weight, resulting in an overall lightweight product. Moreover, we managed to maintain the compressive strength for the concrete samples after replacing 1% of the total aggregate. All these excellent results open a potential avenue for the construction industry to use a composite of recycled EPS and GTR, and all future work needs to now focus on replacing whole or half of the conventional aggregate with the EPS/GTR/SBS composite developed in this work.

## Figures and Tables

**Table 1:** Blend compositions explored in this work.

Sample Code	EPS (wt%)	SBS (wt%)	GTR (wt%)	GTR/EPS Ratio (%)
Melt blend (M)	70	10	20	28.6
Solvent blend (S)	70	0	30	42.8

**Table 2:** Compositions and sample names.

Sample Name	Composite Aggregate Type	Composite Aggregate (wt%)	Cement (wt%)	Coarse Aggregate (wt%)	Fine Aggregate (Sand) (wt%)	Water (wt%)
Control (C1)	-	-	22	-	64	14
10% Sand replacement (M1)	Melt blend	7	22	-	57	14
10% Sand replacement (S1)	Solvent blend	7	22	-	57	14
10% cement replacement (M2)	Melt blend	2	20	-	64	14
10% cement replacement (S2)	Solvent blend	2	20	-	64	14
Concrete control (C2)	-	-	17	42	32	9
1% aggregate replacement (MC3)	Melt blend	1	17	41.5	31.5	9
1% aggregate replacement (SC3)	Solvent blend	1	17	41.5	31.5	9

10% sand replacement shows that 10% sand by weight is being replaced with aggregate and all the calculations in this table is according to the quantity of sand. Similarly, 10% cement replacement represents to the 10% cement replacement by weight is replaced with aggregate and all the calculations are according to the quantity of cement.

**Table 3:** Workability, porosity, and density of fabricated mortar samples.

Type	Sample	Workability (mm)	Bulk Density (g cm^−3^)	Porosity (%)
	Control	115 ± 5	2.032 ± 0.004	8.85 ± 0.18
**Sand replacement**	Solvent blending	105 ± 4	1.816 ± 0.011	17.46 ± 0.10
	Melt blending	107 ± 3	1.828 ± 0.012	17.75 ± 2.65
**Cement replacement**	Solvent blending	110 ± 6	1.966 ± 0.033	9.01 ± 0.24
	Melt blending	108 ± 4	1.947 ± 0.014	9.59 ± 2.26

**Table 4:** Literature analysis of GTR and EPS use as aggregate in mortar and concrete systems.

System	Replacement Details	Compressive Strength Variation After 28 Days (%)	Flexural Strength Variation After 28 Days (%)	Water Absorption Coefficient Variation (%)	Porosity Variation (%)	Ref.
Solvent blend	10% Sand replacement	7.6% increase	18.4% increase	26.6% increase	97.3% up	This work
	10% Cement replacement	22.2% increase	5.26% increase	3.3% increase	100% up	
Melt blend	10% Sand replacement	Equal	15.8% increase	60% increase	1.8% up	
	10% Cement replacement	12.2% increase	Equal	13.3% increase	8.4% up	
Mortar	Sand with 10 wt% GTR	27% drop	-	4% increase	4% increase	[31]
	Sand with 7.5 wt% EPS	26% drop	-	2% increase	3% increase	
	GTR added at 0.6 wt% of total	7% drop	8% drop	14% drop	2% increase	[41]
	EPS added at 0.6 wt% of total	51% drop	53% drop	31% drop	15% increase	
	53% by volume EPS; no sand	86% drop	-	-	-	[30]
	Sand with 30 vol% C black obtained from GTR	40% drop	-	-	-	[42]
	Sand with 10 wt% shoe rubber	22% drop	22% drop	-	-	[43]
	Sand with 10 wt% S.B.R.	27% drop	17% drop	-	-	
	Sand with 60 vol% EPS	65% drop	-	-	-	[44]
	Light weight aggregate with 15% crumb rubber	53% drop	33% drop	-	-	[45]
	All mortar with 25 wt% GTR	-	-	-	40% increase	[46]
	Sand with 40 vol% GTR	27% drop	-	-	-	[47]
	Sand with 20 vol% GTR	40% drop	32% drop	34% increase	-	[48]
Concrete	Cement with 15 vol% EPS	23% drop	19% drop	25% increase	-	[34]
	Sand with 10 wt% GTR	17% drop	7% drop	9% drop	-	[29]
	Coarse gravel with 10 vol% EPS	23% drop	-	-	-	[32]
	Coarse aggregate with 25 vol% GTR	16% drop	20% drop	-	-	[33]
	Coarse aggregate with 16.3 vol% EPS	64% drop	-	-	-	[49]
	Coarse and fine aggregate with 10 vol% GTR	-	14% drop	28% increase	-	[50]
	Coarse gravel aggregate with 10 vol% GTR	33% drop	-	-	-	[51]
	Coarse gravel with 10 vol% GTR	26% drop *	-	-	-	[52]
	Coarse aggregate with 4 wt% GTR	3% drop *	-	-	-	[53]
	Coarse gravel with 10 vol% EPS	7% drop	-	-	-	[54]

*—7 day compressive strength.

## Data Availability

The original contributions presented in the study are included in the article, further inquiries can be directed to the corresponding author.

## References

[1] Bolden J., Abu-Lebdeh T., Fini E. (2013). Utilization of Recycled and Waste Materials in Various Construction Applications. Am. J. Environ. Sci..

[2] Madurwar M.V., Ralegaonkar R.V., Mandavgane S.A. (2013). Application of agro-waste for sustainable construction materials: A review. Constr. Build. Mater..

[3] Awoyera P.O., Adesina A. (2020). Plastic wastes to construction products: Status, limitations and future perspective. Case Stud. Constr. Mater..

[4] Hao H., Bi K., Chen W., Pham T.M., Li J. (2023). Towards next generation design of sustainable, durable, multi-hazard resistant, resilient, and smart civil engineering structures. Eng. Struct..

[5] Huseien G.F., Mirza J., Ismail M., Ghoshal S.K., Hussein A.A. (2017). Geopolymer mortars as sustainable repair material: A comprehensive review. Renew. Sustain. Energy Rev..

[6] Liew K.M., Akbar A. (2020). The recent progress of recycled steel fiber reinforced concrete. Constr. Build. Mater..

[7] Ban Y., Zhi W., Fei M., Liu W., Yu D., Fu T., Qiu R. (2020). Preparation and Performance of Cement Mortar Reinforced by Modified Bamboo Fibers. Polymers.

[8] Kaplan G., Bayraktar O.Y. (2021). The effect of hemp fiber usage on the mechanical and physical properties of cement based mortars. Res. Eng. Struct. Mater..

[9] Mercante I., Alejandrino C., Ojeda J.P., Chini J., Maroto C., Fajardo N. (2018). Mortar and concrete composites with recycled plastic: A review. Sci. Technol. Mater..

[10] Jamshidi A., White G. (2019). Evaluation of Performance and Challenges of Use of Waste Materials in Pavement Construction: A Critical Review. Appl. Sci..

[11] American Chemistry Council 2022 Resin Situation and Trends 2023.

[12] Montagna L.S., Santana R.M.C. (2012). Influence of rubber particle size on properties of recycled thermoplastics containing rubber tyre waste. Plast. Rubber Compos..

[13] Poulakis J.G., Papaspyrides C.D. (1997). Recycling of polypropylene by the dissolution/reprecipitation technique: I. A model study. Resour. Conserv. Recycl..

[14] Carvalho C.H.R., Motta L.A.C. (2019). Study about concrete with recycled expanded polystyrene. Rev. IBRACON Estrut. Mater..

[15] Herki B.A., Khatib J.M. (2016). Valorisation of waste expanded polystyrene in concrete using a novel recycling technique. Eur. J. Environ. Civ. Eng..

[16] Ramli Sulong N.H., Mustapa SA S., Abdul Rashid M.K. (2019). Application of expanded polystyrene (EPS) in buildings and constructions: A review. J. Appl. Polym. Sci..

[17] Assaad J.J., Mikhael C., Hanna R. (2022). Recycling of waste expanded polystyrene concrete in lightweight sandwich panels and structural applications. Clean. Mater..

[18] Menezes I., De Oliveira D., Da Silva S., Brito T., Gachet L., Cecche R. (2018). Study and application of mortars with residues of expanded polystyrene. Int. J. Dev. Res..

[19] Milling A., Mwasha A., Martin H. (2020). Exploring the full replacement of cement with expanded polystyrene (EPS) waste in mortars used for masonry construction. Constr. Build. Mater..

[20] Kibria M.G., Rahaman O., Wahid M.F., Salam A. Effect of Recycled Polystyrene Polymer in Concrete as a coarse aggregate. Proceedings of the Civil and Water Resources Engineering Conference.

[21] Mohajerani A., Burnett L., Smith J.V., Markovski S., Rodwell G., Rahman M.T., Kurmus H., Mirzababaei M., Arulrajah A., Horpibulsuk S. (2020). Recycling waste rubber tyres in construction materials and associated environmental considerations: A review. Resour. Conserv. Recycl..

[22] Narang N., Kaushik M.K., Sharma A., Guleria S.P. (2016). Large Scale Waste Tire Reutilization Practices for GTR in Civil Applications. https://www.researchgate.net/profile/M-Kaushik-2/publication/305755579_Large_scale_waste_tire_reutilization_practices_for_granulated_and_chopped_rubber_Tires_in_Civil_Engineering_Applications/links/579f57bf08ae80bf6ea7b2a4/Large-scale-waste-tire-reutilization-practices-for-granulated-and-chopped-rubber-Tires-in-Civil-Engineering-Applications.pdf.

[23] Tasalloti A., Chiaro G., Murali A., Banasiak L. (2021). Physical and Mechanical Properties of Granulated Rubber Mixed with Granular Soils—A Literature Review. Sustainability.

[24] Nehdi M., Khan A. (2001). Cementitious Composites Containing Recycled Tire Rubber: An Overview of Engineering Properties and Potential Applications. Cem. Concr. Aggreg..

[25] Rigotti D., Dorigato A. (2022). Novel uses of recycled rubber in civil applications. Adv. Ind. Eng. Polym. Res..

[26] Sambucci M., Valente M. (2021). Ground Waste Tire Rubber as a Total Replacement of Natural Aggregates in Concrete Mixes: Application for Lightweight Paving Blocks. Materials.

[27] Fazli A., Rodrigue D. (2020). Recycling Waste Tires into Ground Tire Rubber (GTR)Rubber Compounds: A Review. J. Compos. Sci..

[28] Medina N.F., Garcia R., Hajirasouliha I., Pilakoutas K., Guadagnini M., Raffoul S. (2018). Composites with recycled rubber aggregates: Properties and opportunities in construction. Constr. Build. Mater..

[29] Pietrzak A., Ulewicz M. (2023). Influence of Post-Consumer Waste Thermoplastic Elastomers Obtained from Used Car Floor Mats on Concrete Properties. Materials.

[30] Maaroufi M., Belarbi R., Abahri K., Benmahiddine F. (2021). Full characterization of hygrothermal, mechanical and morphological properties of a recycled expanded polystyrene-based mortar. Constr. Build. Mater..

[31] Pczieczek A., Schackow A., Effting C., Dias T.F., Gomes I.R. (2017). Properties of Mortars containing Tire Rubber Waste and Expanded Polystyrene (EPS). J. Urban Environ. Eng..

[32] Tamut T., Prabhu R., Venkataramana K., Yaragal S.C. (2014). Partial Replacement of Corase Aggregates by Expanded Polystyrene beads in Concrete. Int. J. Res. Eng. Technol..

[33] Bing C., Ning L. (2014). Experimental Research on Properties of Fresh and Hardened Rubberized Concrete. J. Mater. Civ. Eng..

[34] Sadrmomtazi A., Sobhani J., Mirgozar M.A., Najimi M. (2012). Properties of multi-strength grade EPS concrete containing silica fume and rice husk ash. Constr. Build. Mater..

[35] Thorneycroft J., Orr J., Savoikar P., Ball R.J. (2018). Performance of structural concrete with recycled plastic waste as a partial replacement for sand. Constr. Build. Mater..

[36] Binici H., Gemci R., Kaplan H. (2012). Physical and mechanical properties of mortars without cement. Constr. Build. Mater..

[37] Kumar D., Alam M., Sanjayan J. (2023). Experimental and numerical investigation of novel light weight concrete panels made with aerogel and phase change materials. Energy Build..

[38] Kumar D., Alam M., Sanjayan J., Haris M. (2023). Comparative analysis of form-stable phase change material integrated concrete panels for building envelopes. Case Stud. Constr. Mater..

[39] Farouk A.I.B., Hassan N.A., Mahmud M.Z.H., Mirza J., Jaya R.P., Hainin M.R., Yaacob H., Yusoff N.I.M. (2016). Effects of mixture design variables on rubber–bitumen interaction: Properties of dry mixed rubberized asphalt mixture. Mater. Struct..

[40] Chen X., Wu S., Zhou J. (2013). Influence of porosity on compressive and tensile strength of cement mortar. Constr. Build. Mater..

[41] Lanzón M., Cnudde V., De Kock T., Dewanckele J. (2015). Microstructural examination and potential application of rendering mortars made of tire rubber and expanded polystyrene wastes. Constr. Build. Mater..

[42] Zhao J., Huang G., Guo Y., Gupta R., Liu W.V. (2023). Developing thermal insulation cement-based mortars using recycled carbon black derived from scrapped off-the-road tires. Constr. Build. Mater..

[43] Corinaldesi V., Mazzoli A., Moriconi G. (2011). Mechanical behaviour and thermal conductivity of mortars containing waste rubber particles. Mater. Des..

[44] Ferrándiz-Mas V., Bond T., García-Alcocel E., Cheeseman C.R. (2014). Lightweight mortars containing expanded polystyrene and paper sludge ash. Constr. Build. Mater..

[45] Leong G.W., Chin T.M., Mo K.H., Ibrahim Z., Putra A., Othman M.N. (2021). Incorporation of crumb rubber and air-entraining agent in ultra-lightweight cementitious composite: Evaluation of mechanical and acoustic properties. J. Build. Eng..

[46] Corredor-Bedoya A.C., Zoppi R.A., Serpa A.L. (2017). Composites of scrap tire rubber particles and adhesive mortar—Noise insulation potential. Cem. Concr. Compos..

[47] Yang G., Fan Y., Li X., Xu Y. (2023). Influence of rubber powder size and volume fraction on dynamic compressive properties of rubberized mortar. Powder Technol..

[48] Aliabdo A.A., Abd Elmoaty M., AbdElbaset M.M. (2015). Utilization of waste rubber in non-structural applications. Constr. Build. Mater..

[49] Saradhi Babu D., Babu K.G., Wee T.H. (2005). Properties of lightweight expanded polystyrene aggregate concretes containing fly ash. Cem. Concr. Res..

[50] Chaikaew C., Sukontasukkul P., Chaisakulkiet U., Sata V., Chindaprasirt P. (2019). Properties of Concrete Pedestrian Blocks Containing Crumb Rubber from Recycle Waste Tyres Reinforced with Steel Fibres. Case Stud. Constr. Mater..

[51] Turatsinze A., Garros M. (2008). On the modulus of elasticity and strain capacity of Self-Compacting Concrete incorporating rubber aggregates. Resour. Conserv. Recycl..

[52] Raffoul S., Garcia R., Pilakoutas K., Guadagnini M., Medina N.F. (2016). Optimisation of rubberised concrete with high rubber content: An experimental investigation. Constr. Build. Mater..

[53] Nagalakshmi R., Sathiyabama J., Rajendran S., Basha I.A. (2016). Electrochemical study on corrosion inhibition of metals in artificial urine in presence of sodium chloride. Int. J. Chem. Sci..

[54] Topacio A., Marcos M.C.M. (2018). Lightweight interlocking blocks using expanded polystyrene foam as partial replacement to coarse aggregates. The 2018 World Congress on Advances in Civil, Environmental, & Materials Research (ACEM18).

